# Three New Eriophyid Mite Species from China (Acari: Eriophyidae)

**DOI:** 10.3390/insects14020159

**Published:** 2023-02-05

**Authors:** Ke-Xin Hao, Parisa Lotfollahi, Xiao-Feng Xue

**Affiliations:** 1 Department of Entomology, Nanjing Agricultural University, Nanjing 210095, China; 2 Department of Plant Protection, Faculty of Agriculture, Azarbaijan Shahid Madani University, Tabriz P.O. Box 53714-161, Iran

**Keywords:** Eriophyoidea, *Leipothrix*, *Neotegonotus*, *Scolotosus*, taxonomy

## Abstract

**Simple Summary:**

The Eriophyidae is the largest family in the Eriophyoidea, consisting of over 3790 extant species. Eriophyid mites have a patchy distribution worldwide and are concentrated in the temperate regions. In this study, we describe and illustrate three new eriophyid mites from the temperate region of China—*Scolotosus ehretus* **sp. nov.**, *Neotegonotus ulmchangus* **sp. nov.**, and *Leipothrix ventricosis* **sp. nov.** All three new species are vagrant on the lower leaf surface, causing no apparent symptoms to the host plant.

**Abstract:**

Eriophyid mites (Eriophyidae) are strictly phytophagous and are concentrated in Europe, Eastern Asia, Southeast Asia, Western and Eastern North America, Southern India, and New Zealand. South and southwest China are hot spots for eriophyid mite species diversity and endemism. In this study, we describe two new species, *Scolotosus ehretus* **sp. nov.** on *Ehretia acuminata* (Boraginaceae) and *Neotegonotus ulmchangus* **sp. nov.** on *Ulmus changii* (Ulmaceae), from south and southwest China (the Oriental Region), and one new eriophyid mite, *Leipothrix ventricosis* **sp. nov.** on *Hosta ventricosa* (Asparagaceae), from northeast China (the Palearctic Region). All three new eriophyid mite species are distributed in the temperate region of China. We further provided mitochondrial gene (*cox1*, *12S* rRNA) and nuclear gene (*18S* rRNA, *28S* rRNA) sequences for three new species.

## 1. Introduction

Eriophyoid mites are strictly phytophagous [1]. Over 80% of eriophyoid mite species are monophagous [2,3]. Host plants supposedly played key roles in their diversification [4,5]. The Eriophyoidea include three families—Phytoptidae (ca. 160 species), Eriophyidae (ca. 3790 species), and Diptilomiopidae (ca. 450 species) [1,6]. The Eriophyidae can be differentiated from the other two families by having a gnathosoma relatively small and projecting obliquely downwards (a gnathosoma large and abruptly curved near the base in the Diptilomiopidae) and a prodorsal shield without setae *vi* and *ve* (a prodorsal shield has setae *vi* and *ve* in the Phytoptidae) [1]. Although eriophyoid mites have a worldwide distribution, the richness centers differ among the three families—the Eriophyidae are concentrated in Europe, Eastern Asia, Southeast Asia, Western and Eastern North America, Southern India, and New Zealand [4]. South and southwest China are hot spots for eriophyid mite species diversity and endemism [4].

Field surveys of eriophyoid mites have been conducted in China since 1980 [7]. To date, more than 1200 eriophyoid mites have been described in China [8]. Based on a DNA barcoding dataset, the Chinese fauna of eriophyoid mites was inferred, including over 2300 species [3]. To understand the species diversity of eriophyoid mites in China, Xiao-Feng Xue and colleagues have been conducting a long-term field survey since 2002. In this study, we describe and illustrate two new eriophyid mite species (*Scolotosus ehretus* **sp. nov.** and *Neotegonotus ulmchangus* **sp. nov.**) from south and southwest China (the Oriental Region) and one new eriophyid mite species (*Leipothrix ventricosis* **sp. nov.**) from northeast China (the Palearctic Region). All three new eriophyid mite species are distributed in the temperate region of China. We further provided mitochondrial and nuclear gene sequences for three new species.

## 2. Materials and Methods

### 2.1. Taxa Sampling and Morphological Identification

Samples were collected in the field using a hand lens (30×) in China. Mite samples were stored in 96% ethanol at −20 ℃ prior to DNA extraction. Mite specimens were also slide-mounted using Keifer’s Booster and modified Berlese medium [9], but without adding additional fibers, as was suggested by de Lillo et al. [10]. The morphological terminology used herein follows Lindquist [11] and Amrine et al. [1], the internal female genitalia nomenclature follows Chetverikov [12], and the generic classification is made according to Amrine et al. [1] in combination with descriptions of all the published genera after 2003. Specimens were measured following de Lillo et al. [10]. They were examined with the aid of a Zeiss A2 (Carl Zeiss, Gottingen, Germany) research microscope with phase contrast, and semi-schematic drawings were made. Microphotographs were taken with a Zeiss A2 (microphoto camera AxioCam MRc) research microscope with phase contrast or differential interference using 10× eyepieces at 100× oil magnification, connected to a computer using Axiovision (Rel. 4.8) image analysis software. For each species, the holotype female measurements precede the corresponding range for paratypes (given in parentheses). For males, only ranges are given. If no variation was observed among measurements, it will be indicated with an “*”. All measurements are in micrometers (μm) and represent lengths when not otherwise specified. The holotypes and paratypes are deposited in the Arthropod/Mite Collection of the Department of Entomology, Nanjing Agricultural University (NJAU), Jiangsu Province, China [13].

### 2.2. DNA Extraction and Sequencing

Eriophyid mites have limited distinguishable morphological characters, leading to many species complexes [3,14]. To provide more information besides morphological characteristics, we sequenced fragments of two mitochondrial (*cox1*, *12S* rRNA) and two nuclear (*18S* rRNA, *28S* rRNA) genes for three new species (i.e., *Leipothrix ventricosis* sp. nov., *Scolotosus ehretus* sp. nov., *Neotegonotus ulmchangus* sp. nov.) using published PCR primer pairs for each fragment [14]. Genomic DNA was extracted using a DNeasy Blood and Tissue Kit (Qiagen) following a modified protocol [15]. PCR reaction, purification, and sequencing followed Liu et al. [14].

## 3. Results

### Taxonomy

Family Eriophyidae Nalepa

Subfamily Phyllocoptinae Nalepa

Tribe Phyllocoptini Nalepa

Genus *Leipothrix* Keifer

**Figure 1:**
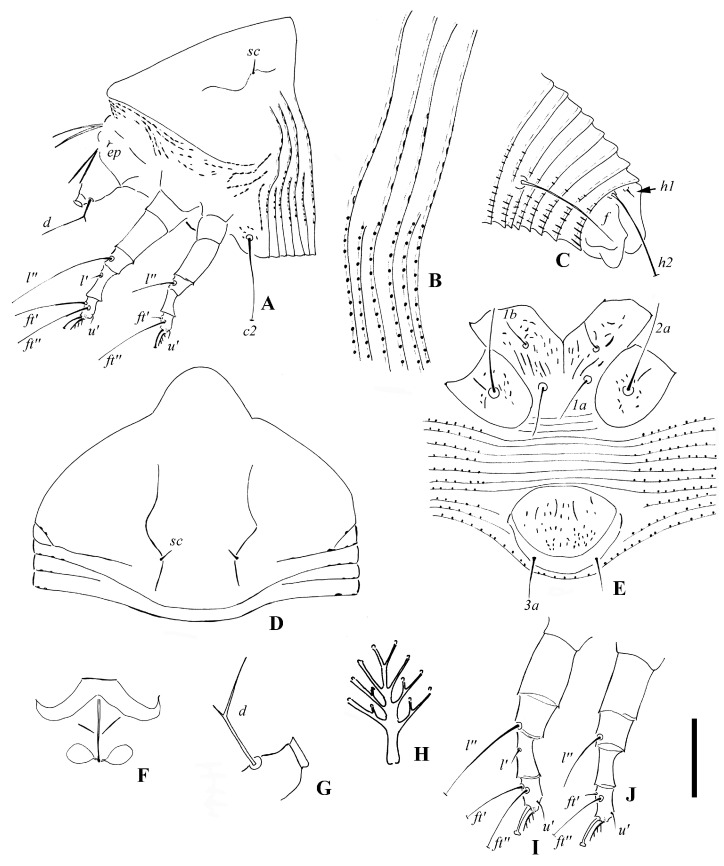
*Leipothrix ventricosis* sp. nov. (**A**) lateral view of anterior part of body; (**B**) lateral microtubercles; (**C**) lateral view of telosoma; (**D**) prodorsal shield; (**E**) female coxigenital area; (**F**) female internal genitalia; (**G**) pedipalp and setae *d*; (**H**) empodium; (**I**) leg І; (**J**) leg II. Scale bar: 23 μm for (**A**,**C**); 19 μm for (**D**–**F**); 15 μm for (**I**,;**J**); 27 μm for (**G**); 2.5 μm for (**B**,**H**) Italic letters depict the abbreviation of setae name.

**Figure 2:**
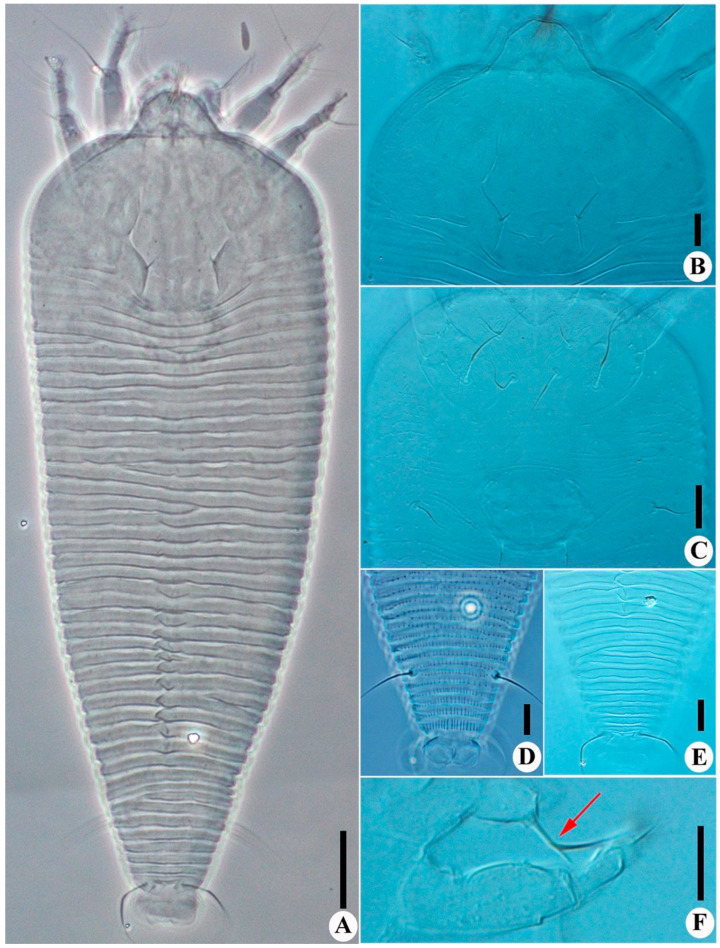
*Leipothrix ventricosis* sp. nov. (**A**) dorsal view; (**B**) prodorsal shield; (**C**) female coxigenital area; (**D**) ventral view of telosoma; (**E**) dorsal view of telosoma; (**F**) dorsal pedipalp genual setae *d*, arrow indicates the branched seta. Scale bar: 20 μm for (**A**); 10 μm for (**B**–**F**).

*Leipothrix ventricosis* sp. nov.

(Figure 1 and Figure 2)

Description. Female (*n* = 8): Body fusiform, 229 (229–246), 81 (81–85) wide, and 70 (69–70) thick; light yellow in color. Gnathosoma 20 (17–20), projecting obliquely downwards, cheliceral stylets 18 (18–20), pedipalp coxal seta (*ep*) 2 (2–3), dorsal pedipalp genual setae (*d*) divided (Figure 1G and Figure 2F), the longer branch 17 (17–20), the shorter branch 3 (2–3), and palp tarsal ventral setae (*v*) absent. Prodorsal shield 61 (58–61), including the frontal lobe, 81 (81–85) wide, frontal lobe broad; median and admedian lines absent, submedian lines incomplete on the posterior 2/3. Scapular tubercles ahead of the rear shield margin, setae *sc* 5 (4–5), 19 (19–21) apart, projecting centrad. Coxigenital region with 10 * semiannuli between coxal plates and genitalia, smooth; coxal plates with dashes and short lines, anterolateral setae on coxisternum І (*1b*) 9 (8–10), 18 (18–19) apart; proximal setae on coxisternum І (*1a*) 16 (11–16), 11 (11–12) apart; proximal setae on coxisternum ІІ (*2a*) 23 (20–23), 32 (32–34) apart. Prosternal apodeme present, 10 (10–11). Leg І 29 (29–30), femur 10 *, basiventral femoral setae (*bv*) absent; genu 5 *, antaxial genual setae (*l’’*) 30 (29–30); tibia 7 (7–8), paraxial tibial setae (*l’*) 4 *, located at basal 1/3; tarsus 5*, paraxial fastigial tarsal setae *ft’* 18 (16–18), antaxial fastigial tarsal setae *ft’’* 22 (20–22), setae *u’* 4 (4–5); tarsal empodium (*em*) 6 (5–6), simple, 4-rayed, tarsal solenidion (*ω*) 6 *, knobbed. Leg ІІ 28 (28–29), femur 10 (9–10), basiventral femoral setae (*bv*) absent; genu 5 *, antaxial genual setae (*l’’*) 11 (8–11); tibia 6 *; tarsus 5 (5–6), paraxial fastigial tarsal setae *ft’* 3 *, antaxial fastigial tarsal setae *ft’’* 21 (18–21), setae *u’* 3 (3–4); tarsal empodium (*em*) 6 (5–6), simple, 4-rayed, tarsal solenidion (*ω*) 6 (5–6), knobbed. Opisthosoma dorsally with 42 (42–43) semiannuli, with elliptical microtubercles at the lateral side (Figure 1B,D), with three ridges, the middorsal ridge smooth and ending in a furrow; ventrally with 73 (71–75) semiannuli, with round microtubercles, except the posterior 15–17 semiannuli with elliptical to linear microtubercles. Setae *c2* 11 (11–14), on ventral semiannulus 10 (10–12), 58 (58–62) apart; setae *d* 15 (13–15), on ventral semiannulus 29 (28–29), 35 (35–37) apart; setae *e* 10 (10–11), on ventral semiannulus 52 (50–52), 17 (17–20) apart; setae *f* 26 (24–26), 25 (25–26) apart, on the 6th ventral semiannulus from the rear; setae *h1* 4 (3–4), setae *h2* 48 (46–48). Female genitalia 20 (20–21), 26 (25–26) wide, coverflap with granules and short lines, setae *3a* 11 (9–11), 15 (15–17) apart. Internal genitalia: spermathecae ovoid, oriented posterolaterad; spermathecal tubes relatively short as long as 1/4 of spermathecal length; transverse genital apodeme trapezoidal.

Male: Not found.

Type material. Holotype, female (slide number NJAUH92.1; marked Holotype), found on *Hosta ventricosa* Stearn (Asparagaceae), Heilongjiang Forest Botanical Garden, Harbin city, Heilongjiang province, China, 45°62′87″N, 127°21′79″E, elevation 151 m, 27 July 2018, coll. Liang-Fei Yao and Yue Yin, deposited as a slide-mounted specimen in the Arthropod/Mite Collection of the Department of Entomology, NJAU. Paratypes, seven females on seven slides (slide number NJAU H92.2–NJAU H92.8; marked paratypes), from *Hosta ventricosa* Stearn (Asparagaceae), same details as the holotype, deposited as slide-mounted specimens in the Arthropod/Mite Collection of the Department of Entomology, NJAU.

GenBank accession numbers. MZ279892 (*18S* rRNA); MZ288929, MZ326514 (*28S* rRNA); MZ255319 (*12S* rRNA).

Relation to the host plant. Vagrant on the lower leaf surface. No apparent symptom of the host plant was observed.

Etymology. The specific designation *ventricosis* is derived from the species name of the host plant, *ventricosa*, changing postfix -*a* to -*is*; feminine in gender.

Differential diagnosis. The new species is morphologically similar to *Leipothrix juniperensis* Xue and Yin, 2020 [16], by the presence of divided dorsal pedipalp genual setae (*d*), dorsal opisthosoma with three ridges, and coxal plates with granules and short lines, but can be differentiated by admedian lines absent from the prodorsal shield (admedian lines present in *L. juniperensis*), female genital coverflap with granules and short lines (female genital coverflap with 6 to 8 ribs in *L. juniperensis*).

Tribe Tegonotini Bagdasarian

Genus *Scolotosus* Flechtmann and Keifer

**Figure 3:**
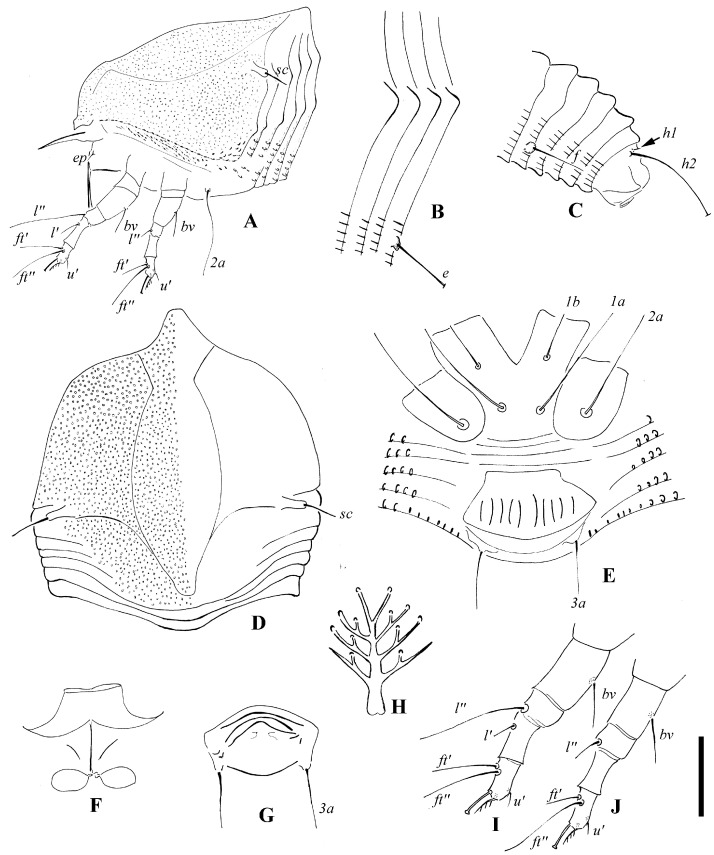
*Scolotosus ehretus* sp. nov. (**A**) lateral view of the anterior part of the body; (**B**) lateral microtubercles; (**C**) lateral view of the telosoma; (**D**) prodorsal shield; (**E**) female coxigenital area; (**F**) female internal genitalia; (**G**) male external genitalia; (**H**) empodium; (**I**) leg І; (**J**) leg II. Scale bar: 18 μm for (**A**,**C**); 15 μm for (**D**–**G**); 12 μm for (**I**,**J**); 3 μm for (**B**,**H**). Italic letters depict the abbreviation of setae name.

**Figure 4:**
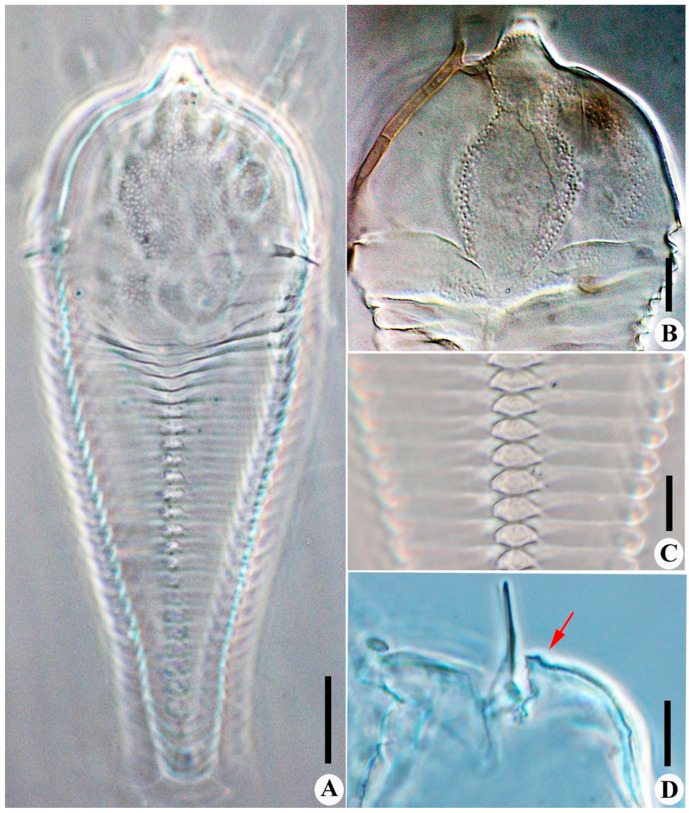
*Scolotosus ehretus* sp. nov. (**A**) dorsal view; (**B**) prodorsal shield; (**C**) doral annuli; (**D**) lateral view of the gnathosoma; the arrow indicates the lateral view of the frontal lobe. Scale bar: 20 μm for (**A**); 10 μm for (**B**–**D**).

*Scolotosus ehretus* sp. nov.

(Figure 3 and Figure 4)

Description. Female (*n* = 10): Body fusiform, 167 (150–167), 58 (58–64) wide, and 40 (36–40) thick; light yellow in color. Gnathosoma 17 (17–18), projecting obliquely downwards, cheliceral stylets 14 (14–15), pedipalp coxal seta (*ep*) 3 (3–4), dorsal pedipalp genual setae (*d*) 6 (6–7), palp tarsal ventral setae (*v*) absent. Prodorsal shield 64 (58–64), including the frontal lobe, is 58 (58–63) wide, with the frontal lobe 5 (5–6) being broad (Figure 3A,D and Figure 4A,B,D); median and submedian lines are absent, admedian lines are concave at the anterior 1/4, forming a vase-shape; many pits are present on the prodorsal shield (Figure 3D and Figure 4A,B). Scapular tubercles ahead of rear shield margin, 5 (4–5), setae *sc* 7 (7–8), 44 (44–45) apart, projecting lateral. Coxigenital region with 4 * smooth semiannuli between coxal plates and genitalia; coxal plates smooth, anterolateral setae on coxisternum І (*1b*) 6 (6–7), 12 (10–12) apart; proximal setae on coxisternum І (*1a*) 16 (13–16), 7 (7–8) apart; proximal setae on coxisternum ІІ (*2a*) 24 (20–24), 18 (18–22) apart. Prosternal apodeme is absent. Leg І 29 (29–31), femur 10 (9–10), basiventral femoral setae (*bv*) 7 (7–8); genu 5 (4–5), antaxial genual setae (*l’’*) 17 (17–18); tibia 6 (5–6), paraxial tibial setae (*l’*) 4 (3–4), located at basal 1/3; tarsus 6 (5–6), paraxial fastigial tarsal setae *ft’* 13 (13–16), antaxial fastigial tarsal setae *ft’’* 18 (16–18), setae *u’* 2 (2–3); tarsal empodium (*em*) 5 (5–6), simple, 4-rayed, tarsal solenidion (*ω*) 5 (5–6), knobbed. Leg ІІ 25 (25–26), femur 9 (9–10), basiventral femoral setae (*bv*) 5 (5–6); genu 4 (4–5), antaxial genual setae (*l’’*) 5 (4–5); tibia 5 *; tarsus 5 (4–5), paraxial fastigial tarsal setae *ft’* 5 (4–5), antaxial fastigial tarsal setae *ft’’* 17 (17–18), setae *u’* 3 (3–4); tarsal empodium (*em*) 5 *, simple, 4-rayed, tarsal solenidion (*ω*) 5 *, knobbed. Opisthosoma dorsally with 28 (28–29) semiannuli, smooth, with three ridges, middorsal ridge as long as subdorsal ridges extended on the whole dorsal opisthosoma; and ventrally with 50 (47–50) semiannuli, with elliptical to linear microtubercles. Setae *c2* 8 (8–9), on ventral semiannulus 7 *, 45 (43–45) apart; setae *d* 35 (35–40), on ventral semiannulus 16 (16–17), 30 (30–31) apart; setae *e* 6 (6–8), on ventral semiannulus 29 (28–29), 13 (12–13) apart; setae *f* 16 (15–16), 16 (16–18) apart, on the 5th ventral semiannulus from rear; setae *h1* 4 *, setae *h2* 61 (61–63). Female genitalia 14 (14–15), 22 (22–23) wide, coverflap with 10 longitudinal striae, setae *3a* 13 (13–15), 15 (15–16) apart. Internal genitalia: spermathecae ovoid, oriented posterolaterad; spermathecal tubes relatively short, as long as 1/5 of spermathecal length; transverse genital apodeme trapezoidal.

Male (*n* = 2): Body fusiform, 136–145, 49–53 wide; light yellow in color. Gnathosoma 16–17, projecting obliquely downwards, cheliceral stylets 10 *, pedipalp coxal seta (*ep*) 2 *, dorsal pedipalp genual setae (*d*) 5 *, palp tarsal ventral setae (*v*) absent. Prodorsal shield 53–57, including the frontal lobe, is 49–53 wide. Scapular tubercles ahead of the rear shield margin, setae *sc* 5–6, 43–45 apart, projecting lateral. Coxigenital region with 4 smooth semiannuli between coxal plates and genitalia, coxal plates smooth, anterolateral setae on coxisternum І (*1b*) 7 *, 14 * apart; proximal setae on coxisternum І (*1a*) 15–16, 7–9 apart; proximal setae on coxisternum ІІ (*2a*) 20 *, 21–22 apart. Prosternal apodeme is absent. Leg І 25–26, femur 9 *, basiventral femoral setae (*bv*) 5 *; genu 4 *, antaxial genual setae (*l’’*) 15–19; tibia 5 *, paraxial tibial setae (*l’*) 3 *, located at basal 1/3; tarsus 5 *, paraxial fastigial tarsal setae *ft’* 10 *, antaxial fastigial tarsal setae *ft’’* 16 *, setae *u’* 3 *; tarsal empodium (*em*) 5 *, simple, 4-rayed, tarsal solenidion (*ω*) 5 *, knobbed. Leg ІІ 24–26, femur 8 *, basiventral femoral setae (*bv*) 5 *; genu 4 *, antaxial genual setae (*l’’*) 5–6; tibia 5 *; tarsus 5 *, paraxial fastigial tarsal setae *ft’* 5–6, antaxial fastigial tarsal setae *ft’’* 16–17, setae *u’* 3 *; tarsal empodium (*em*) 5 *, simple, 4-rayed, tarsal solenidion (*ω*) 5 *, knobbed. Opisthosoma dorsally with 27 * semiannuli, smooth; ventrally with 49–50 semiannuli, with elliptical to linear microtubercles. Setae *c2* 10–11, on ventral semiannulus 7 *, 44–45 apart; setae *d* 30–32, on ventral semiannulus 16 *, 30 * apart; setae *e* 6 *, on ventral semiannulus 29 *, 14–15 apart; setae *f* 14 *, 15 * apart, on the 5th ventral semiannulus from the rear; setae *h1* 3 *, setae *h2* 56–58. Male genitalia are 10 *, 17 * wide, and setae *3a* 10–11, 15 * apart.

Type material. Holotype, female (slide number NJAUGX54.1; marked Holotype), found on *Ehretia acuminata* R. Brown (Boraginaceae), Guilin city, Guangxi Zhuang Autonomous Region, China, 25°26′89″N, 110°31′21″E, elevation 164 m, 29 August 2017, coll. Liang-Fei Yao and Yue Yin, deposited as a slide-mounted specimen in the Arthropod/Mite Collection of the Department of Entomology, NJAU. Paratypes, nine females on nine slides and two males on two slides (slide number NJAU GX54.2–NJAU GX54.12; marked paratypes), from *Ehretia acuminata* R. Br. (Boraginaceae), same details as the holotype, were deposited as slide-mounted specimens in the Arthropod/Mite Collection of the Department of Entomology, NJAU.

GenBank accession numbers. MZ279974 (*18S* rRNA); MZ289015, MZ326597 (*28S* rRNA); MZ274841 (*cox1*); MZ255373 (*12S* rRNA).

Relation to the host plant. Vagrant on the lower leaf surface. No apparent symptom to the host plant was observed.

Etymology. The specific designation *ehretus* is derived from the generic name of the host plant, *Ehretia*, changing the postfix -*ia* to -*us*, which is masculine in gender.

Differential diagnosis. Only two *Scolotosus* species were reported worldwide (Table 1). The new species is morphologically similar to *Scolotosus centrolobii*, Flechtmann and Keifer, 2010 [17], with the presence of many pits on the prodorsal shield and scapular tubercles located laterally, but can be differentiated by smooth coxal plates (coxal plates with short lines in *S. centrolobii*) and smooth dorsal annuli (dorsal annuli with granules in *S. centrolobii*). The new species is also similar to *Scolotosus hartfordi* Flechtmann and de Queiroz, 2010 [17], by the presence of many pits on the prodorsal shield, smooth coxal plates, and scapular tubercles located laterally, but can be differentiated by a female genital coverflap with ridges (smooth coverflap in *S. hartfordi*), and an empodium 4-rayed (empodium 5-rayed in *S. hartfordi*).

Genus *Neotegonotus* Newkirk and Keifer

**Figure 5:**
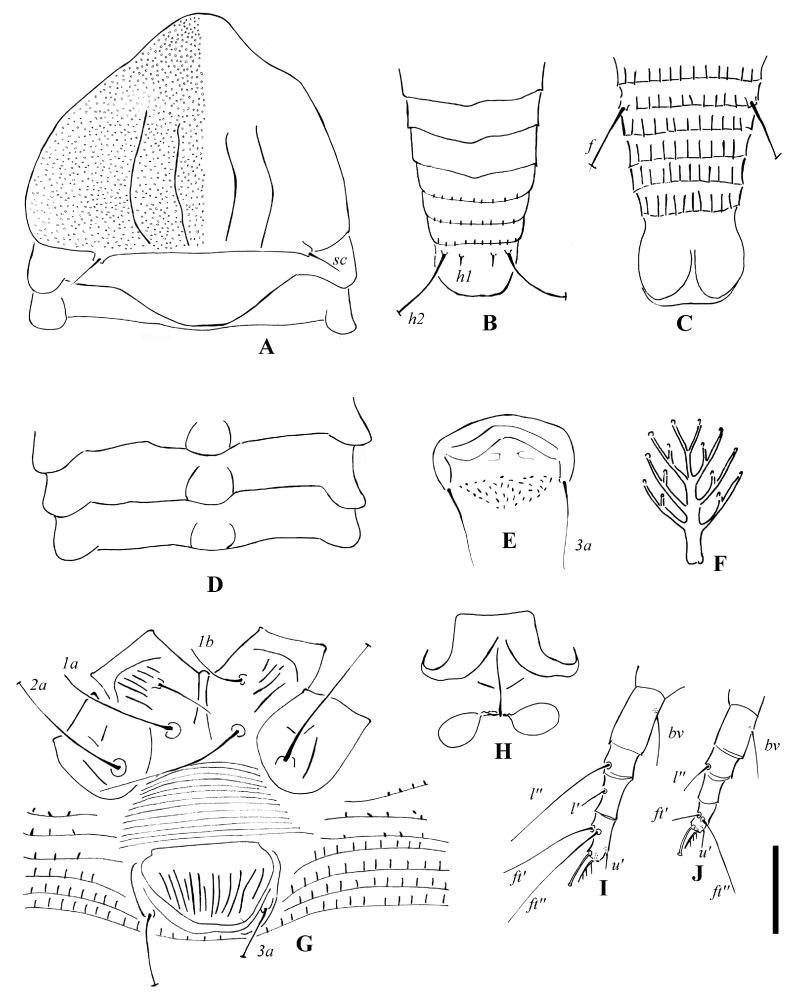
*Neotegonotus ulmchangus* sp. nov. (**A**) prodorsal shield; (**B**) dorsal view of telosoma; (**C**) ventral view of telosoma; (**D**) dorsal annuli; (**E**) male external genitalia; (**F**) empodium; (**G**) female coxigenital area; (**H**) female internal genitalia; (**I**) leg І; (**J**) leg II. Scale bar: 19 μm for A, B, C, D; 13 μm for E, G, H; 15 μm for I, J; 3 μm for F. Italic letters depict the abbreviation of setae name.

**Figure 6:**
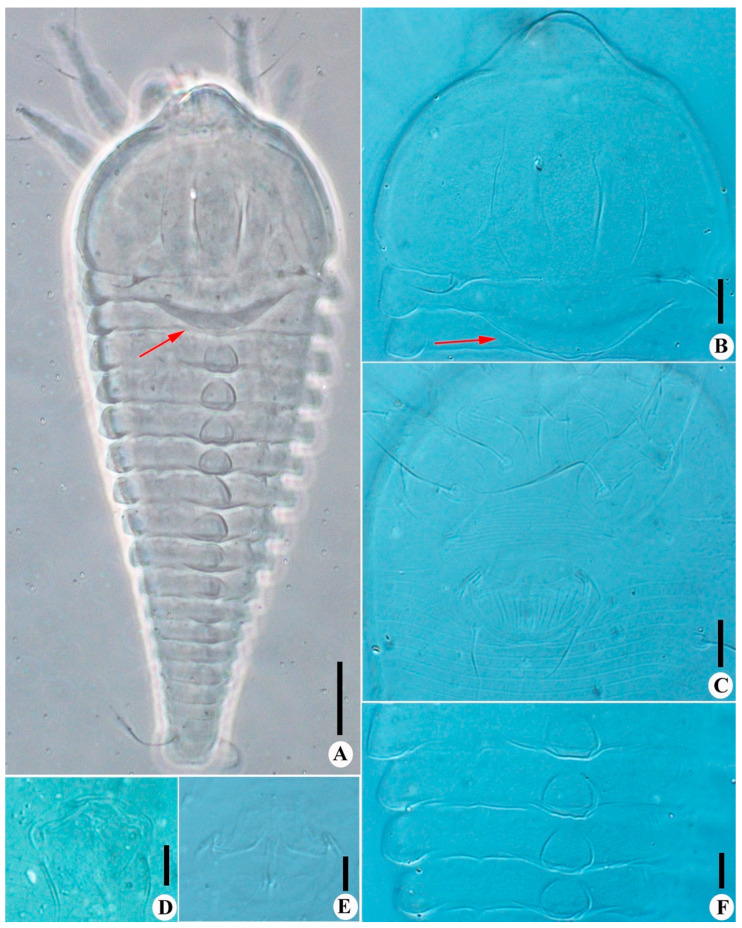
*Neotegonotus ulmchangus* sp. nov. (**A**) dorsal view; (**B**) prodorsal shield; (**C**) female coxigenital area; (**D**) male genitalia; (**E**) female internal genitalia; (**F**) dorsal annuli. Scale bar: 20 μm for A; 10 μm for B, C, F; 5 μm for D, E. Arrows indicate the first dorsal annuli broad, projecting the posterior.

*Neotegonotus ulmchangus* sp. nov.

(Figure 5 and Figure 6)

Description. Female (*n* = 8, dorsal view): Body fusiform, 179 (166–179), 67 (67–68) wide; light yellow in color. Gnathosoma 18 (16–18), projecting obliquely downwards, cheliceral stylets 15 (15–16), pedipalp coxal seta (*ep*) 3 (2–3), dorsal pedipalp genual setae (*d*) 6 (6–7), palp tarsal ventral setae (*v*) absent. Prodorsal shield 50 (50–51), including the frontal lobe, is 68 (67–70) wide, the frontal lobe 5 (5–6) is broad (Figure 5A and Figure 6B); the median line is absent, the admedian and submedian lines are incomplete, they are parallel at the posterior 2/3; many pits are present on the prodorsal shield (Figure 5A and Figure 6B). Scapular tubercles on the rear shield margin, setae *sc* 10 (9–10), 44 (42–44) apart, projecting the posterior. Coxigenital region with 13–14 smooth semiannuli between coxal plates and genitalia; coxal plates with short lines, anterolateral setae on coxisternum І (*1b*) 9 (8–9), 13 (13–14) apart; proximal setae on coxisternum І (*1a*) 14 (14–15), 9 (9–10) apart; proximal setae on coxisternum ІІ (*2a*) 30 (27–30), 24 (24–27) apart. Prosternal apodeme is present, 9 (9–10). Leg І 29 (29–31), femur 10 (9–10), basiventral femoral setae (*bv*) 10 (9–10); genu 5 (4–5), antaxial genual setae (*l’’*) 20 (19–20); tibia 6 (5–6), paraxial tibial setae (*l’*) 5 (4–5), located at basal 1/3; tarsus 6 (5–6), paraxial fastigial tarsal setae *ft’* 18 (18–19), antaxial fastigial tarsal setae *ft’’* 20 *, setae *u’* 3 (3–4); tarsal empodium (*em*) 5 (5–6), simple, 4-rayed, tarsal solenidion (*ω*) 5 (5–6), knobbed. Leg ІІ 25 (25–26), femur 9 *, basiventral femoral setae (*bv*) 10 (8–10); genu 5 (4–5), antaxial genual setae (*l’’*) 4 (4–5); tibia 5 *; tarsus 5 (5–6), paraxial fastigial tarsal setae *ft’* 5 (4–5), antaxial fastigial tarsal setae *ft’’* 21 (17–21), setae *u’* 3 (3–4); tarsal empodium (*em*) 5 *, simple, 4-rayed, tarsal solenidion (*ω*) 5 *, knobbed. Opisthosoma dorsally with 16 (16–17) semiannuli, smooth, the first dorsal annuli broad, projecting posterior (Figure 5A and Figure 6A,B); with three ridges, the middorsal ridge as long as the subdorsal ridges; ventrally with 56 (53–56) semiannuli, with elliptical to linear microtubercles. Setae *c2* 18 (17–19), on ventral semiannulus 11 (10–11), 55 (55–60) apart; setae *d* 35 (35–39), on ventral semiannulus 19 (18–19), 33 (33–36) apart; setae *e* 11 (8–11), on ventral semiannulus 34 (32–34), 14 (14–16) apart; setae *f* 20 (18–20), 18 (16–18) apart, on the 5th ventral semiannulus from rear; setae *h1* 2 *, setae *h2* 55 (54–58). Female genitalia 15 (14–15), 20 (20–21) wide, coverflap with 13–14 longitudinal striae, setae *3a* 15 (12–15), 17 (17–18) apart. Internal genitalia: spermathecae ovoid, oriented posterolaterad; spermathecal tubes relatively short, as long as 1/3 of spermathecal length; transverse genital apodeme trapezoidal.

Male (*n* = 3, dorsal view): Body fusiform, 140–145, 51–52 wide; light yellow in color. Gnathosoma 15–16, projecting obliquely downwards, cheliceral stylets 13 *, pedipalp coxal seta (*ep*) 2 *, dorsal pedipalp genual setae (*d*) 6 *, palp tarsal ventral setae (*v*) absent. Prodorsal shield 45–46, including the frontal lobe, is 51–53 wide. Scapular tubercles on the rear shield margin, setae *sc* 10–11, 35–36 apart, projecting posterior. Coxigenital region with 13 smooth semiannuli between coxal plates and genitalia, coxal plates with short lines, anterolateral setae on coxisternum І (*1b*) 8 *, 13 * apart; proximal setae on coxisternum І (*1a*) 15–16, 9–10 apart; proximal setae on coxisternum ІІ (*2a*) 24–27, 20–21 apart. Prosternal apodeme is present, 9 (9–10). Leg І 25–26, femur 9 *, basiventral femoral setae (*bv*) 9–10; genu 4 *, antaxial genual setae (*l’’*) 18–19; tibia 5 *, paraxial tibial setae (*l’*) 4 *, located at basal 1/3; tarsus 5 *, paraxial fastigial tarsal setae *ft’* 17 *, antaxial fastigial tarsal setae *ft’’* 20 *, setae *u’* 3 *; tarsal empodium (*em*) 5–6, simple, 4-rayed, tarsal solenidion (*ω*) 5 *, knobbed. Leg ІІ 24–26, femur 9 *, basiventral femoral setae (*bv*) 8 *; genu 4 *, antaxial genual setae (*l’’*) 5–6; tibia 5 *; tarsus 5 *, paraxial fastigial tarsal setae *ft’* 5–6, antaxial fastigial tarsal setae *ft’’* 17–18, setae *u’* 3 *; tarsal empodium (*em*) 5 *, simple, 4-rayed, tarsal solenidion (*ω*) 5 *, knobbed. Opisthosoma dorsally with 17 * semiannuli, smooth; ventrally with 49–50 semiannuli, with elliptical to linear microtubercles. Setae *c2* 17–18, on ventral semiannulus 10 *, 45–46 apart; setae *d* 30–32, on ventral semiannulus 18 *, 35 * apart; setae *e* 8 *, on ventral semiannulus 27 *, 14–15 apart; setae *f* 15–16, 15 * apart, on the 5th ventral semiannulus from the rear; setae *h1* 2 *, setae *h2* 57–60. Male genitalia 11 *, 16 * wide, setae *3a* 10–11, 15 * apart.

Type material. Holotype, female (slide number NJAUFJ27.1; marked Holotype), found on *Ulmus changii* W.C. Cheng (Ulmaceae), Wuyi mountain, Fujian province, China, 27°40′28″N, 118°2′37″E, elevation 251 m, 21 August 2017, coll. Liang-Fei Yao and Yue Yin, deposited as a slide-mounted specimen in the Arthropod/Mite Collection of the Department of Entomology, NJAU. Paratypes, seven females on seven slides and three males on three slides (slide number NJAUFJ27.2–NJAUFJ27.11; marked paratypes), from *Ulmus changii* W.C. Cheng (Ulmaceae), with the same details as the holotype, deposited as slide-mounted specimens in the Arthropod/Mite Collection of the Department of Entomology, NJAU.

GenBank accession numbers. MZ279799 (*18S* rRNA); MZ288836, MZ326418 (*28S* rRNA); MZ255260 (*12S* rRNA).

Relation to the host plant. Vagrant on the lower leaf surface. No apparent symptom of the host plant was observed.

Etymology. The specific designation *ulmchangus* is derived from the combination of the host plant name, *Ulmus changii*, by sdeleting -*us* from the genus name and changing the postfix -*ii* to -*us* in the species name; masculine in gender.

Differential diagnosis. Six *Neotegonotus* species were described worldwide (Table 2). The new species is morphologically similar to *N. pengensis*, Meyer, 1990 [18], by the presence of many pits on prodorsal shield, coxal plates with short lines, and female genital coverflap with many longitudinal striae, but can be differentiated by dorsal annuli smooth (dorsal annuli with microtubercles in *N. pengensis*), admedian and submedian lines incomplete (admedian lines complete, submedian lines absent in *N. pengensis*), and empodium 4-rayed (empodium 5-rayed in *N. pengensis*).

Key to species of *Neotegonotus*

1. Dorsal annuli smooth……….…….…………….………...……………….……………..2

- Dorsal annuli with microtubercles……………………….………………..……………3

2. Coxal area smooth, empodium 5-rayed….…*N. indicus* Mondal and Chakrabarti, 1982

- Coxal area with short lines, empodium 4-rayed………....….…*N. ulmchangus* **sp. nov.**

3. Coxal area smooth……………………………………………. *N. shangsiensis* Wei, 2003

- Coxal area with granules or short lines........................................................................…4

4. Female genital coverflap with two rows of striae…...*N. sycamori* Abou–Awad, 1984

- Female genital coverflap with one row of striae………………………………………5

5. Setae *h1* present, empodium 5-rayed………………………………………………..…6

- Setae *h1* absent, empodium 4-rayed…………………....…. *N. alopebaccae* Meyer, 1990

6. Prodorsal shield with lined granules, admedian lines complete, submedian lines absent.……………………………………………….……………*N. pengensis* Meyer, 1990

- Prodorsal shield without lined granules, submedian lines complete, admedian lines absent……………………………………………………………*N. fastigatus* (Nalepa 1892)

## 4. Discussion

In this study, we described and illustrated three new eriophyoid mite species from three genera (*Leipothrix*, *Scolotosus*, and *Neotegonotus*) in China. All three new species belong to the subfamily Phyllocoptinae (Eriophyidae), based on the distinct morphological characters—body shape fusiform; gnathosoma relatively small, projecting obliquely downwards; with legs having the typical segmentation; dorsal annuli broader than the ventral annuli (Amrine et al., 2003) [1]. However, the monophyly of Phyllocoptinae was rejected by molecular studies [5,28,29] as well as the non-monophyly of two genera, *Leipothrix* and *Neotegonotus* [5]. It should be noted that the phylogenetic position of *L. ventricosis* **sp. nov.** (named *Leipothrix* sp. H92 in Figure 2 of reference [5]) was inferred to be nested with three *Paraepitrimerus* species [5]. *L. ventricosis* **sp. nov.** is morphologically similar to species in the genus *Paraepitrimerus* [30] by exhibiting femur setae absent, an opisthosoma with three ridges, and scapular setae and tubercles ahead of the rear shield margin, but can be differentiated distinctly by dorsal pedipalp genual setae (*d*) divided, which is a generic character of *Leipothrix* [1]. Two *Neotegonotus* species, *N. ulmchangus* **sp. nov.** (named *Neotegonotus* sp. FJ27 in Figure 2 of reference [5]), and *N. shangsiensis*, failed to be grouped in the analysis of reference [5]. By contrast, *N. ulmchangus* **sp. nov.** was inferred to be closer to species in the genera *Aculus*, *Abacarus*, and *Shevtchenkella* [5]. *S. ehretus* **sp. nov.** (named *Scolotosus* sp. GX54 in Figure 2 of reference [5]) was nested within species from genera *Tegolophus* and *Aculus* [5]. Therefore, it is likely that their generic characteristics should be reconsidered in future studies. All three new species are distributed in the temperate region of China. The species diversity and endemism of eriophyid mites peak in temperate regions [4]. The south and southwest mountains of China are hot spots for eriophyid mite species diversity and endemism [4], reflecting potential “museums” and “cradles”. Given the over 1200 eriophyoid mite species described in China [8] and suspected 2300 species for the eriophyoid mite fauna of China [3], more field surveys should be conducted, especially in the mountains of southwest China, to unveil the species diversity of eriophyoid mites.

The *Leipothrix* is among the most species-rich genera in the Eriophyidae, including more than 50 named species (personal data of X.-F.X.). Eighteen *Leipothrix* species have been reported in China. Most of them (13/18) were distributed in the temperate region, while the remaining species were distributed in the tropical region. *S. ehretus* **sp. nov.** is one of three *Scolotosus* species reported in the temperate region of China. The other two *Scolotosus* species, *S. centrolobii* Flechtmann and Keifer, 2010, and *S. hartfordi* Flechtmann and de Queiroz, 2010, were distributed in the temperate region of Brazil [17]. The genus *Neotegonotus* holds seven species (including *N. ulmchangus* **sp. nov.,** Table 2), of which *N. fastigatus* and *N. sycamori* have a cosmopolitan distribution [18,20,21,22,23,26,27]. All species in three genera (*Leipothrix*, *Scolotosus*, and *Neotegonotus*) have high (100%) host plant specificity; no alternative host plants were reported for each eriophyoid mite species.

Eriophyid mites have simplified morphological characteristics (e.g., only two pairs of legs, reduced setae on the opisthosoma and legs, ringed opisthosoma) when compared with other mite species [1]. Two fossil species dating back to the Triassic are morphologically similar to extant species [31]. It is likely that the eriophyoid mites were in morphological stasis during long-term evolution, leading to a potential species complex [3,14,32]. *COI* barcodes were recently explored in eriophyoid mite delimitation, enabling the discrimination of 99% of eriophyoid mite species [3]. Besides morphological characteristics, we provide a *COI* barcode for *Scolotosus ehretus* **sp. nov.** However, we failed to sequence the *COI* barcode for the other two species. We further provided mitochondrial (*12S* rRNA) and nuclear gene (*18S* rRNA, *28S* rRNA) sequences for *Leipothrix ventricosis* **sp. nov.**, *Scolotosus ehretus* **sp. nov.**, *Neotegonotus ulmchangus* **sp. nov.** We suggested a combination of molecular sequences and morphological characters in the description of new eriophyoid mite species.

## Figures and Tables

**Table 1:** Gross comparison of some important traits among *Scolotosus ehretus* sp. nov., *S. centrolobii* Flechtmann and Keifer, 2010, and *S. hartfordi* Flechtmann and de Queiroz, 2010.

Characters	*S. ehretus* sp. nov.	*S. centrolobii*	*S. hartfordi*
Dorsal semiannuli number	28–29	26	26
Ventral semiannuli number	47–50	26	26–30
Number of semiannuli between coxal plates and genital coverflap	4	4	4
Number of empodium I rays	4	5	5
Setae *sc* length	7–8	9	8–10
Setae *c2* length	8–9	12	7–10
Setae *d* length	35–40	38	24–37
Setae *e* length	6–8	9	6–9
Setae *f* length	15–16	9	16–18
Setae *h1* length	4	3	3
Setae *3a* length	13–15	10	6–11

**Table 2:** List of *Neotegonotus* species.

Species	Hosts	Distribution	Relation to Host
*N. alopebaccae* Meyer, 1990 [18]	*Diospyros mespiliformis* Hochstetter ex A. P. de Candolle (Ebenaceae)	South Africa	Vagrant
*N. fastigatus* (Nalepa 1892) [19]	*Acer campestre* Linnaeus (Sapindaceae)	Palearctic [20,21], USA [22], India [23]	Erineum or vagrant
*N. indicus* Mondal and Chakrabarti, 1982 [24]	*Ficus benghalensis* Linnaeus (Moraceae)	India	Apical shoots brownish and stunted
*N. pengensis* Meyer, 1990 [18]	*Ficus capreifolia* Delile (Moraceae)	South Africa	Vagrant
*N. shangsiensis* Wei, 2003 [25]	*Acronychia pedunculata* (Linnaeus) Miquel (Rutaceae)	China	Vagrant
*N. sycamori* Abou–Awad, 1984 [26,27]	*Ficus sycomorus* Linnaeus (Moraceae)	Egypt, South Africa [18]	Vagrant
*N. ulmchangus* **sp. nov.**	*Ulmus changii* W.C. Cheng (Ulmaceae)	China	Vagrant

## Data Availability

All data is available in this paper. All sequences were deposited in the GenBank under accession numbers of MZ255260, MZ255373, MZ255319, MZ274841, MZ279799, MZ279892, MZ279974, MZ288836, MZ288929, MZ289015, MZ326418, MZ326514, and MZ326597.
